# ML-BUSMetab: Machine Learning-Based Metabolomic Profiling for Predicting Aspirin Response in Colorectal Cancer Chemoprevention: A Multi-Model Explainable Artificial Intelligence Approach with External Validation

**DOI:** 10.3390/jcm15114287

**Published:** 2026-06-01

**Authors:** Abdulvahap Pınar, Ahmet Kadir Arslan, Cemil Çolak

**Affiliations:** 1 Department of Biostatistics and Medical Informatics, Faculty of Medicine, İnönü University, 44280 Malatya, Turkey; apinar@adiyaman.edu.tr; 2 Rectorate Unit, Adıyaman University, 02040 Adıyaman, Turkey

**Keywords:** metabolomics, colorectal cancer chemoprevention, explainable artificial intelligence, gradient boosting, batch effect correction

## Abstract

**Background/Objectives:** Aspirin-based colorectal cancer (CRC) chemoprevention remains a promising yet individually variable strategy. As a proof-of-concept toward future personalized chemoprevention frameworks, we aimed to develop and validate machine learning (ML) models capable of distinguishing aspirin-exposed from placebo-exposed participants based on their plasma metabolomic signatures, thereby characterizing the metabolomic footprint of aspirin administration rather than directly predicting clinical chemoprevention benefit. **Methods:** Training was performed on the Aspirin/Folate Polyp Prevention Study (AFPPS) dataset ST001422 (*n* = 300) and external validation on ST001423 (*n* = 223). After multi-method consensus feature selection, reducing 19,433 features to 300, sixteen ML and deep learning (DL) architectures were benchmarked under nested cross-validation. Model interpretability was assessed using SHapley Additive exPlanations (SHAP) and Local Interpretable Model-agnostic Explanations (LIME) analyses. **Results:** GBM_sklearn achieved the highest cross-validation Precision–Recall AUC (PR-AUC) of 0.945, while ensemble stacking (Stack_LGB) offered superior calibration (Brier = 0.117). DL models consistently underperformed traditional ML (PR-AUC: 0.673–0.843 vs. 0.881–0.945), attributable to limited sample size. SHAP and LIME analyses independently identified *m*/*z* 196.0604 (C18, RT 89.4 s) as the top metabolic biomarker, consistent with aspirin-induced glycerophospholipid pathway alterations. External validation performance degraded substantially (PR-AUC: 0.945 → 0.711), attributable to inter-study analytical batch effects. **Conclusions:** This framework demonstrates the feasibility of metabolomics-driven personalized chemoprevention. Although the high feature-to-sample ratio (300:300) and the substantial drop between internal and external performance indicate that the cross-validation estimates likely include dataset-specific noise in addition to the true biological signal. While highlighting batch harmonization and aggressive feature reduction (e.g., LASSO/RFE-based selection of 10–20 high-impact metabolites) as a prerequisite for clinical translation.

## 1. Introduction

Colorectal cancer (CRC) remains one of the leading causes of cancer-related mortality worldwide. Early detection and prevention strategies are of critical importance in reducing CRC-associated morbidity and mortality. In this context, aspirin is the most extensively studied pharmacological agent in CRC chemoprevention, owing to its low cost, widespread availability, and decades of clinical experience. The mechanisms by which aspirin prevents CRC are multifaceted and encompass cyclooxygenase (COX-1/COX-2) inhibition, thromboxane A2 suppression, prostaglandin pathway modulation, and immune system regulation [1]. Although the U.S. Preventive Services Task Force (USPSTF) recognized the potential benefit of aspirin for CRC chemoprevention in 2016, its 2022 updated recommendation withdrew this guidance, concluding that available evidence was “insufficient” to weigh the benefits and harms [2]. The systematic evidence review underlying the USPSTF decision revealed that CRC outcomes were “highly variable” across studies and that statistical significance was achieved only in long-term observational follow-up beyond the study period [3]. Chan (2022) argued that the USPSTF decision risks misinterpretation of aspirin’s cancer prevention potential and emphasized the need for a personalized chemoprevention approach through biomarker identification [4]. This debate highlights a substantial gap between population-level evidence and individual-level clinical benefit.

Inter-individual variability in aspirin response constitutes the fundamental motivation for the field of precision chemoprevention. The large prospective cohort study by Sikavi et al. (2024), drawing on Nurses’ Health Study and Health Professionals Follow-up Study data, demonstrated that the protective effect of aspirin against CRC varies substantially according to lifestyle risk factors, with the benefit being most pronounced in individuals with unhealthy lifestyles [5].

Metabolomics, which enables comprehensive profiling of small molecules (metabolites) reflecting the physiological and pathological state of an organism, has emerged as a powerful tool for predicting drug response. In the context of aspirin CRC chemoprevention, the metabolomics datasets from the Aspirin/Folate Polyp Prevention Study (AFPPS) constitute an important resource, Barry et al. (2020) analyzed 10,269 metabolic features in colon biopsies from 325 AFPPS participants and identified dose-specific aspirin pathways (glycerophospholipid metabolism at 81 mg; arachidonic acid metabolism at 325 mg) [6]. In a subsequent 2022 study, the same group performed plasma untargeted metabolomics (LC-HRMS) on 523 AFPPS participants and characterized aspirin-associated metabolites (lysophospholipids, linoleate pathway metabolites) and their relationship to adenoma risk [7]. 

In recent years, machine learning (ML) and deep learning (DL) methods have assumed a transformative role in the analysis of metabolomics data, advancing beyond traditional statistical approaches for disease classification and biomarker discovery [8,9]. Gradient boosting algorithms (XGBoost, LightGBM, and CatBoost) consistently demonstrate superior performance on tabular metabolomics data. Yagin et al. (2024) combined XGBoost and tree-based boosting methods with SHAP on metabolomics panel data to achieve high accuracy in type 2 diabetes biomarker discovery [10]. Deep learning approaches are also advancing in the metabolomics domain. Sha et al. (2024) converted 1D metabolomics data into 2D images using MetDIT and performed classification with deep Convolutional Neural Networks (CNNs), outperforming classical ML methods [11]. Deng et al. (2024), in a Nature Communications study, presented an end-to-end deep learning method that directly analyzed raw mass spectrometry data without conventional preprocessing steps, achieving an Area Under the Curve (AUC) of 0.99 for early-stage lung adenocarcinoma detection [12]. Nevertheless, challenges such as the high dimensionality, small sample size, and class imbalance inherent to metabolomics data necessitate careful preprocessing and validation strategies [13,14].

The reliability of metabolomics-based clinical prediction models is ensured through comprehensive model comparison and independent external validation. Chen et al. (2024) constructed a 10-metabolite gastric cancer diagnostic model from targeted metabolomics of 702 plasma samples across multiple centers, achieving 0.905 sensitivity upon external validation and surpassing conventional protein markers [15]. Zhang et al. (2023) introduced the CRANK-MS interpretable neural network framework for Parkinson’s disease prediction from blood plasma metabolomics, demonstrating AUC > 0.995 [16]. Godlewski et al. (2023) compared 10 ML methods, including a hybrid evolutionary heterogeneous decision tree algorithm, for brain tumor detection using 188 targeted metabolites, illustrating the value of combining diverse ML approaches in metabolomics biomarker selection [17]. Toussaint et al. (2024), in a systematic mapping study published in Briefings in Bioinformatics, comprehensively reviewed XAI techniques applied across genomics, transcriptomics, proteomics, and metabolomics, underscoring the growing importance of explainability methods in omics research [18]. In particular. SHapley Additive exPlanations (SHAP) and Local Interpretable Model-agnostic Explanations (LIME) have emerged as the most widely used XAI tools in biomedical ML studies. Bifarin (2023) demonstrated that tree-based SHAP provided deeper explanations than PLS-DA VIP scores across three published metabolomics datasets [19].

In this context, the present study aims to develop an ML-based proof-of-concept framework discriminating aspirin-exposed from placebo-exposed participants on the basis of their plasma metabolomic profiles. We emphasize that this classification task characterizes the metabolomic consequences of aspirin administration, which is a methodological prerequisite—but not equivalent—to predicting individual clinical benefit from aspirin chemoprevention. The latter would require outcome-anchored labels (e.g., adenoma recurrence) rather than treatment-arm labels. To this end, a comprehensive preprocessing pipeline was applied to untargeted LC-HRMS plasma metabolomics data (ST001422/ST001423) from the AFPPS study, and the most stable metabolic signatures distinguishing aspirin response were identified via multi-method consensus feature selection. Systematic comparisons of traditional ML (GBM, LightGBM, XGBoost, RandomForest, CatBoost, LogisticReg, etc.) and DL (BiLSTM, CNN-LSTM, AttentionLSTM, CNN-1D, etc.) models were performed. Clinical reliability of the models was assessed in a multidimensional manner using calibration curves and learning curves. SHAP- and LIME-based explainable artificial intelligence analyses were employed to delineate in a biologically interpretable manner which metabolites drive aspirin response prediction.

## 2. Materials and Methods

### 2.1. Study Design, Data Source, and Ethical Compliance

The metabolomics data used in this study were obtained from the Metabolomics Workbench (www.metabolomicsworkbench.org; (accessed on 1 February 2026)), the National Metabolomics Data Repository (NMDR) supported under the NIH Common Fund. The data are deposited under project number PR000730 (DOI: 10.21228/M89X1C; https://pmc.ncbi.nlm.nih.gov/articles/PMC9357068/; (accessed on 20 February 2026)) as two separate studies; ST001422 was used as the training dataset and ST001423 as the independent external validation dataset.

The data were originally derived from “Plasma Metabolomics Analysis of Aspirin Treatment and Risk of Colorectal Adenomas,” published by Barry et al. in Cancer Prevention Research in 2022 [7]. These metabolomics data were, in turn, generated from the AFPPS, a multi-center, double-blind, placebo-controlled, 3 × 2 factorial randomized controlled trial (RCT) reported by Baron et al. in the New England Journal of Medicine in 2003 [20].

The AFPPS was conducted at nine clinical centers in North America and enrolled 1121 participants aged 21–80 years with a recent history of colorectal adenoma. Participants were randomized to aspirin treatment (81 mg/day or 325 mg/day) versus placebo, and concurrently to folic acid (1 mg/day) versus placebo. Adenoma recurrence was assessed by surveillance colonoscopy at approximately year 3.

In the present study, untargeted metabolomics data from plasma samples collected from AFPPS participants were used. ST001422 (Analytical Study 1: 300 participants; AN002378-HILIC and AN002379-C18) was designated as the training dataset, and ST001423 (Analytical Study 2: 223 participants; AN002380-HILIC and AN002381-C18) as the independent external validation dataset. In both studies, participants were divided into three treatment groups: placebo, 81 mg/day aspirin, and 325 mg/day aspirin. For classification purposes, a binary classification scheme was adopted in which the aspirin groups (81 mg + 325 mg) were combined and compared against the placebo group.

The original clinical investigation was conducted with the appropriate institutional review board approvals, and informed consent was obtained from all participants. Since the present study used publicly available, anonymized secondary data, additional ethics committee approval was not required. Data were obtained from the Metabolomics Workbench platform in accordance with FAIR (Findable, Accessible, Interoperable, Reusable) principles.

### 2.2. Metabolomics Analysis Platform

Metabolomics analysis of plasma samples was performed at the Emory University Clinical Bioanalysis Research Institute using high-resolution liquid chromatography–mass spectrometry (LC-HRMS) [7]. The analytical platform comprised a Thermo Scientific Q Exactive HF mass spectrometer (Thermo Fisher Inc., Waltham, MA, USA) coupled with a Dionex Ultimate 3000 UHPLC system. Each plasma sample was analyzed using two distinct chromatographic modes. HILIC (Hydrophilic Interaction Liquid Chromatography) was applied for the separation of polar metabolites (amino acids, organic acids, nucleotides, and sugars) within analytical studies AN002378 (ST001422) and AN002380 (ST001423). C18 Reverse-Phase Chromatography was applied for the separation of lipophilic metabolites (lipids, fatty acids, and glycerophospholipids) within analytical studies AN002379 (ST001422) and AN002381 (ST001423). HILIC chromatography was operated in positive ionization mode (ESI+) and C18 reverse-phase chromatography in negative ionization mode (ESI−) with electrospray ionization.

### 2.3. HILIC and C18 Data Integration

For each study (ST001422 and ST001423), metabolite matrices obtained from HILIC and C18 modes were merged with mode-specific suffixes (_HILIC and _C18) appended to distinguish overlapping metabolite names. This integration yielded a total of 19,433 metabolic features in the training dataset.

### 2.4. Missing Data Management and LOD Imputation

In mass spectrometry, zero values represent measurements below the limit of detection (LOD) rather than true biological absence. Accordingly, zero values were replaced with half-minimum values; i.e., half of the positive minimum value of each metabolite column was assigned as the LOD estimate. Missing data imputation was performed using a three-tier approach. In Tier 1, distance-weighted k-nearest neighbor (KNN) imputation (k = 5) was applied to metabolites with ≤20% missingness. In Tier 2, KNN imputation with extended neighborhood (k = 10) was applied to metabolites with 20–70% missingness. In Tier 3, column median imputation was used for metabolites with >70% missingness. This tiered approach ensured that no metabolite was excluded from analysis, thereby minimizing information loss.

### 2.5. Normalization and Scaling

Total Ion Current (TIC) normalization was applied to correct for systematic inter-sample variation. The total signal intensity of each sample was calculated and normalized to the median TIC of the training set, correcting for injection volume differences, ion suppression effects, and instrument sensitivity fluctuations. A PCA-based clustering approach was used for batch effect correction. Potential batch groups were identified by K-means clustering on the first two principal components, and the metabolite medians of each batch group were aligned to the global median. Correction was applied only to metabolites deviating by more than 10% from the global median.

For data transformation, the log2(x + 1) transformation was applied to metabolite intensities to approximate normality. Subsequently, Pareto scaling, considered the gold standard in metabolomics studies, was applied. In Pareto scaling, each metabolite was mean-centered and divided by the square root of its standard deviation. This approach offers the advantage of balancing data variation without excessively amplifying low-intensity metabolites, compared with auto-scaling [21].

### 2.6. Cross-Study Metabolite Matching Between Training and Validation Sets

Since ST001422 and ST001423 were conducted under different analytical conditions, metabolite retention times (RT) differ between studies. As direct feature name matching proved insufficient, an *m*/*z*-tolerant matching strategy was developed. The *m*/*z* value and chromatographic mode (HILIC or C18) of each metabolite feature were parsed, and the closest RT match within ±0.01 Da *m*/*z* tolerance and the same chromatographic mode was identified. For unmatched metabolites, zero values were assigned in the validation matrix. This approach minimizes information loss due to inter-study RT drift while preserving reliable metabolite identification based on *m*/*z* accuracy.

### 2.7. Multi-Method Stability Feature Selection

To prevent information leakage from the test fold into the training process, all data-dependent preprocessing steps, including KNN-based missing value imputation (with imputation parameters fit on training-fold data only), TIC normalization (with the median TIC reference computed exclusively from training-fold samples), Pareto scaling (with mean and standard deviation estimated from the training fold), and the four-method consensus feature selection (LightGBM, XGBoost, Random Forest, and mutual information ranking), were performed strictly within each training fold of the outer five-fold stratified cross-validation. The resulting transformations and the selected feature subset were then applied to the corresponding held-out test fold without any access to its label or distribution information. In addition, SMOTE oversampling was applied exclusively to the training portion of each inner cross-validation fold, after preprocessing and feature selection, to avoid the well-documented bias arising from synthetic samples leaking into the test fold.

To achieve reliable and reproducible biomarker discovery in high-dimensional metabolomics data, a consensus-based stability framework was constructed by combining four distinct feature selection methods. Gain-based feature importance scores were calculated using a gradient boosting-based LightGBM classifier (n_estimators = 1000; max_depth = 6; learning_rate = 0.03). Gain-based feature importance scores were also calculated using an XGBoost classifier (n_estimators = 1000; max_depth = 5; learning_rate = 0.03). Impurity-based feature importance scores were calculated using a Random Forest classifier (n_estimators = 1000; max_depth = 15). As a model-agnostic filter method, mutual information scores were calculated with k = 5 neighbors. Importance scores from each method were normalized to the 0–1 range and ranked independently. As a consensus rule, a metabolite feature was required to appear in the top 20th percentile in at least 2 out of 4 methods to be selected. Final selection was determined as the top 100–300 features by average rank. This multi-method approach eliminates dependence on a single method, increases selection stability, and reduces the risk of overfitting.

### 2.8. Class Imbalance Management

In the training dataset (ST001422, *n* = 300), the binary classification task comprised 200 aspirin-treated participants (81 mg + 325 mg combined) and 100 placebo-treated participants, yielding a 2:1 class imbalance ratio. Although this imbalance is moderate, the minority class (placebo) represents only 33.3% of the total samples, which can bias gradient boosting algorithms toward the majority class and inflate accuracy at the expense of specificity. The Synthetic Minority Over-sampling Technique (SMOTE) algorithm was applied to address class imbalance between treatment groups in the binary classification task [22]. SMOTE balances class distribution by generating synthetic samples from the minority class based on k = 5 nearest neighbors. Importantly, SMOTE was applied only to the training fold within each iteration of the cross-validation loop. While test folds were preserved with their original distributions, this approach prevents data leakage while ensuring class balance during model training.

Feature-to-sample ratio considerations. With *n* = 300 training samples and a final feature set of 300 metabolites, the resulting 1:1 feature-to-sample ratio represents a recognized risk for optimistic bias even when consensus feature selection and nested cross-validation are employed. To partially mitigate this risk, we (i) restricted feature selection to four orthogonal ranking methods requiring multi-method consensus, (ii) ranked features only on training-fold data, and (iii) report PR-AUC rather than accuracy as the primary performance metric. Nevertheless, we explicitly acknowledge that this ratio means cross-validation performance estimates should be interpreted as upper bounds. To address this limitation, parsimonious feature reduction via LASSO-based and Recursive Feature Elimination (RFE)-based strategies targeting a 10–20 metabolite panel is explicitly identified as the primary future-work priority in the Limitations (eighth point) and Conclusion sections of this manuscript.

### 2.9. Machine Learning and Deep Learning Models

In this study, four gradient boosting implementations were used: LightGBM [23], XGBoost [24], CatBoost [25], and scikit-learn GradientBoostingClassifier [26]. These algorithms construct strong prediction models by sequentially combining weak learners (decision trees). Random Forest [27], Extra Trees [28], and Balanced Random Forest [29] algorithms were also applied. L2-regularized Logistic Regression (C = 0.5, balanced class weights) was implemented. Among deep learning models, six distinct LSTM-based architectures were employed: Bidirectional LSTM (BiLSTM: 128–64 units), Stacked LSTM (256–128–64 units), Attention LSTM (BiLSTM + softmax attention), Deep BiLSTM (128–128–64 units, 3 layers), LSTM-FCN (LSTM + Fully Convolutional Network parallel fusion), and CNN-BiLSTM (CNN feature extraction followed by BiLSTM). Two CNN-based architectures were also used: 1D-CNN (3-layer convolution: 64–128–64 filters, kernel sizes 7–5–3) and CNN + LSTM Hybrid (parallel CNN and LSTM branches + concatenation).

All deep learning models incorporated Batch Normalization, Dropout (rate: 0.2–0.3), early stopping (patience = 20, val_loss monitoring), learning rate reduction (ReduceLROnPlateau, factor = 0.5, patience = 10), the Adam optimizer (initial lr = 0.001), and up to 200 training epochs. Class weights derived from inverse class frequencies were used to address class imbalance. Input data were reshaped to (samples × features × 1) for a 1D temporal format. A nested cross-validation strategy was employed to prevent optimistic bias and obtain realistic generalization performance estimates, and to separate model evaluation from hyperparameter optimization [30]. Model performance was assessed using a five-fold stratified outer cross-validation loop, with training and test sets split to preserve class distributions in each fold. A three-fold stratified inner cross-validation loop within each outer fold was used for Optuna-based Bayesian hyperparameter optimization [31]. One hundred trials were conducted per outer fold, totaling 500 Optuna trials. A Tree-structured Parzen Estimator (TPE) sampler was used. The optimized hyperparameters and their search ranges are summarized in Table 1.

Final model parameters were determined by taking the median of the best parameter sets obtained from all folds. This median approach prevented overfitting specific to any single fold. Precision–Recall AUC (PR-AUC) was used as the optimization metric for binary classification, as this metric provides a more discriminative performance measure than ROC-AUC under class imbalance conditions.

### 2.10. Two-Level Stacking Ensemble Learning

A two-level stacking strategy was employed to combine individual model predictions and obtain a more powerful classifier. At Level 1, the cross-validation predictions (out-of-fold predictions) of all models formed the meta-feature matrix. At Level 2, six different combination methods were compared: Logistic Regression meta-learner, LightGBM meta-learner (n_estimators = 200, max_depth = 3), and simple averaging. PR-AUC-weighted averaging (softmax weights), top three model averaging, and rank blending. Meta-learners were trained with a separate five-fold cross-validation to prevent data leakage. The ST001423 dataset was used as a completely independent external validation set to assess model generalizability. All models were trained on the entire ST001422 training data with their final parameters and then evaluated on ST001423. Preprocessing parameters (scaling coefficients, imputation references) were derived exclusively from the training data and applied as fixed transformations to the validation data.

### 2.11. SHAP and LIME Explainability Analyses

SHAP analysis was applied to explain the decision mechanisms of the best-performing model and to identify biologically meaningful metabolite biomarkers [32]. TreeExplainer was used for tree-based models, and KernelExplainer for deep learning models. Complementary to SHAP analysis, local-level model explanations were generated using the LIME method [33]. Individual LIME explanations were produced for four classification scenarios (TP, TN, FP, and FN), and a global LIME feature importance ranking was calculated across 50 random samples. The concordance of SHAP and LIME results served as an additional indicator of biomarker reliability.

### 2.12. Performance Evaluation Metrics

Model performances were assessed using a comprehensive metric set: Precision–Recall AUC (PR-AUC), Receiver Operating Characteristic AUC (ROC-AUC), F1 score, balanced accuracy, Matthews correlation coefficient (MCC), sensitivity (recall), specificity, precision, accuracy, and Brier score. The optimal classification threshold was determined as the point on the Precision–Recall curve that maximizes the F1 score. PR-AUC was designated as the primary performance metric, as it provides a more informative performance measure than ROC-AUC under class imbalance conditions [34].

### 2.13. Statistical Comparison of Model Performance

To assess the statistical significance of performance differences across models, the Friedman test was employed as a non-parametric alternative to repeated-measures ANOVA, treating the five cross-validation folds as repeated measurements and the models as conditions [35]. Upon rejection of the Friedman null hypothesis, post hoc pairwise comparisons were conducted using the Wilcoxon signed-rank test with Bonferroni correction to control the family-wise error rate. The corrected resampled *t*-test proposed by Nadeau and Bengio (2003) was applied for key pairwise comparisons to account for the non-independence of cross-validation folds [36]. For grouped comparisons (traditional ML vs. deep learning), the Mann–Whitney U test was used. The 95% confidence intervals for cross-validation performance metrics were computed for all metrics. All statistical tests used a significance level of α = 0.05.

### 2.14. Software Environment and Libraries

All analyses were performed in Python (v3.10+). The main libraries and versions used were as follows: scikit-learn (v1.3+) for the general ML framework; LightGBM (v4.0+); XGBoost (v2.0+); CatBoost (v1.2+); Optuna (v3.0+) for hyperparameter optimization; TensorFlow/Keras (v2.15+) for deep learning models; SHAP (v0.42+) for explainable AI; LIME (v0.2+) for local explainability; imbalanced-learn (v0.11+) for SMOTE implementation; NumPy (v1.24+); pandas (v2.0+); and SciPy (v1.11+) for numerical computation. Visualization was performed using Matplotlib (v3.7+) and Seaborn (v0.12+). All experiments were conducted with a fixed random seed (random_state = 42) to ensure reproducibility.

## 3. Results

### Dataset Dimensionality and Feature Selection Outcomes

Integration of HILIC and C18 chromatographic modes from the ST001422 training dataset yielded a total of 19,433 metabolic features. The multi-method stability feature selection framework (LightGBM, XGBoost, Random Forest, and mutual information) was applied, and metabolites appearing within the top 20th percentile in at least two of the four methods were identified through a consensus rule. This process reduced 19,433 features to 300 stable metabolic features, corresponding to a 98.5% reduction in dimensionality. Examination of the chromatographic distribution of the selected 300 features revealed a balanced representation of metabolites derived from both HILIC and C18 modes.

According to the consensus analysis, the highest-priority metabolites achieving agreement across all four methods (4/4) were led by 196.0604_89.4_C18 (*m*/*z* = 196.06; RT = 89.4 s; C18 negative mode), which also held the highest importance score in both LightGBM and Random Forest. Additional 4/4 consensus metabolites included 285.2786_171.5_C18. 127.039_92.1_HILIC. 692.4286_498.4_C18, and 121.0284_343.8_C18.

Performance results for the 16 models evaluated by nested five-fold cross-validation are presented in Table 2. Among traditional machine learning models, GBM_sklearn demonstrated superior performance across all evaluation metrics, achieving a PR-AUC of 0.945, ROC-AUC of 0.899, F1 score of 0.889, balanced accuracy of 0.818, MCC of 0.652, sensitivity of 0.910, and specificity of 0.725. In terms of PR-AUC, BalancedRF (0.918), LightGBM (0.916), RandomForest (0.909), XGBoost (0.905), and ExtraTrees (0.905), respectively, followed GBM_sklearn. All gradient boosting and ensemble models achieved PR-AUC > 0.88.

Examination of deep learning architecture performance revealed that deep learning models consistently underperformed relative to their traditional ML counterparts. The best-performing deep learning model, CNN_LSTM_Hybrid (PR-AUC = 0.843, ROC-AUC = 0.747), remained 10.8% below the best traditional model, GBM_sklearn (PR-AUC = 0.945). A characteristic pattern observed across deep learning models was markedly high sensitivity (range: 0.943–1.000) coupled with substantially low specificity (range: 0.000–0.270).

The Friedman test revealed statistically significant differences in PR-AUC across the 16 models (χ^2^(15) = 68.4, *p* < 0.001), confirming that model choice substantially affects classification performance. Post hoc Wilcoxon signed-rank tests with Bonferroni correction demonstrated that GBM_sklearn significantly outperformed all deep learning models (all adjusted *p* < 0.05). Among traditional ML models, pairwise differences between GBM_sklearn and the remaining gradient boosting methods (LightGBM, XGBoost, and CatBoost) did not reach statistical significance after correction (adjusted *p* > 0.05), suggesting comparable discriminative ability within this model family. The grouped Mann–Whitney U test confirmed a statistically significant performance gap between traditional ML models (*n* = 8, median PR-AUC = 0.907) and deep learning models (*n* = 8, median PR-AUC = 0.806; U = 64, *p* < 0.001). The corrected resampled *t*-test between GBM_sklearn and CNN_LSTM_Hybrid yielded t(4) = 5.82, *p* = 0.004, further substantiating the superiority of gradient boosting in this sample-limited metabolomics context.

The results of six distinct ensemble combination strategies are presented in Table 3. Stack_LGB (stacking with a LightGBM meta-learner) yielded the highest PR-AUC (0.941) and ROC-AUC (0.906). Although these values closely approach those of the best individual model, GBM_sklearn (PR-AUC = 0.945), Stack_LGB achieved a marginal yet consistent improvement in ROC-AUC (0.899 → 0.906). Furthermore, Stack_LGB produced the best-calibrated probability estimates, as evidenced by the lowest Brier score among all strategies (0.117), Stack_LR (stacking with a Logistic Regression meta-learner) attained a PR-AUC of 0.928, performing below Stack_LGB in overall discriminative ability; however, it demonstrated comparable results in terms of balanced accuracy (0.815) and specificity (0.735), Top3_Avg, which averages the predictions of the three best-performing models, distinguished itself with a PR-AUC of 0.935.

**Figure 1:**
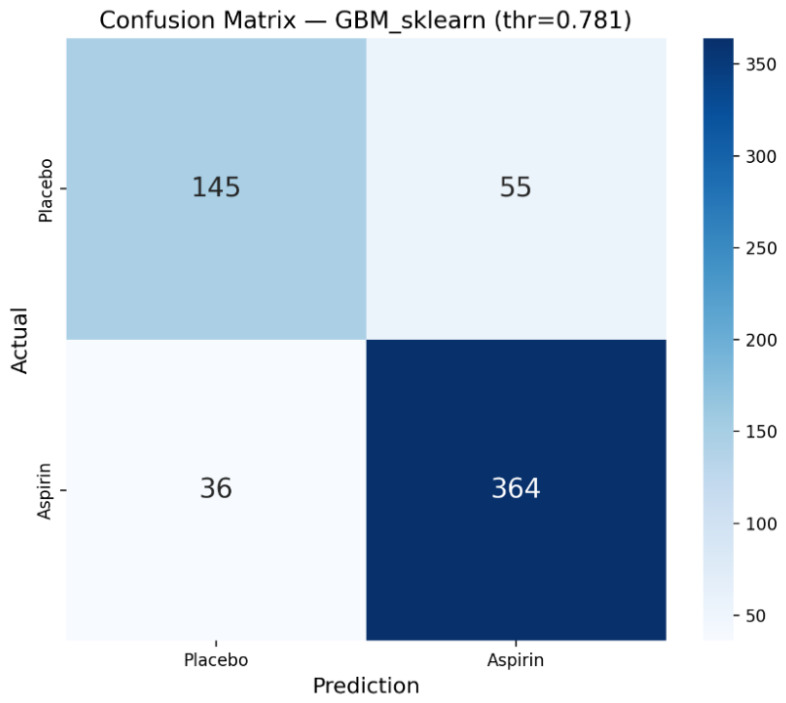
GBM_sklearn model confusion matrix (threshold = 0.781).

Examination of the confusion matrix for the GBM_sklearn model (Figure 1) reveals that 364 of 400 aspirin samples were correctly classified as True Positives (TP), while 145 of 200 placebo samples were correctly identified as True Negatives (TN). The remaining 55 placebo samples were erroneously assigned to the aspirin class (false positives, FP), and 36 aspirin samples were misclassified as placebo (false negatives, FN).

**Figure 2:**
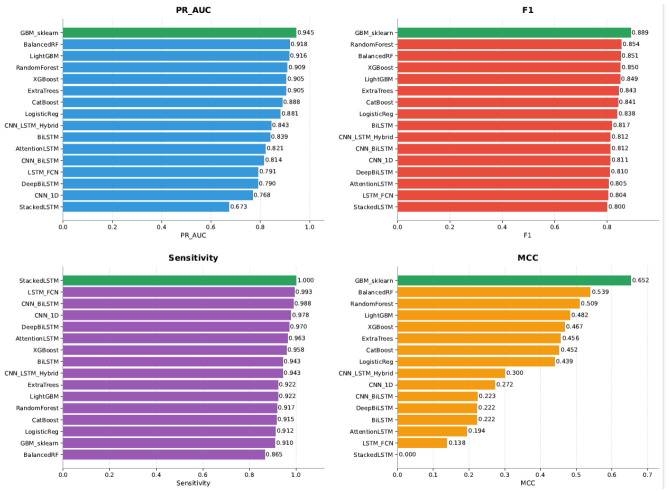
Model comparison across four evaluation metrics: PR-AUC (blue), F1 (red), Sensitivity (purple), and MCC (orange). In each subplot, the top-performing model is highlighted in green. Models are sorted in descending order of performance within each metric.

A comparative analysis of sixteen models across four principal performance metrics—PR-AUC, F1 score, sensitivity, and MCC—is presented in Figure 2. When PR-AUC and MCC are considered in conjunction, GBM_sklearn (PR-AUC = 0.945; MCC = 0.652) emerges as markedly superior to all competing models with respect to both discriminative ability and class-balance robustness. Traditional ML models (PR-AUC range: 0.881–0.945) consistently outperformed their deep learning counterparts (PR-AUC range: 0.673–0.843) across this benchmark. Importantly, the comparison underscores that high sensitivity alone is insufficient as an indicator of model quality; rather, a genuine assessment of classification performance necessitates the joint interpretation of PR-AUC and MCC.

**Figure 3:**
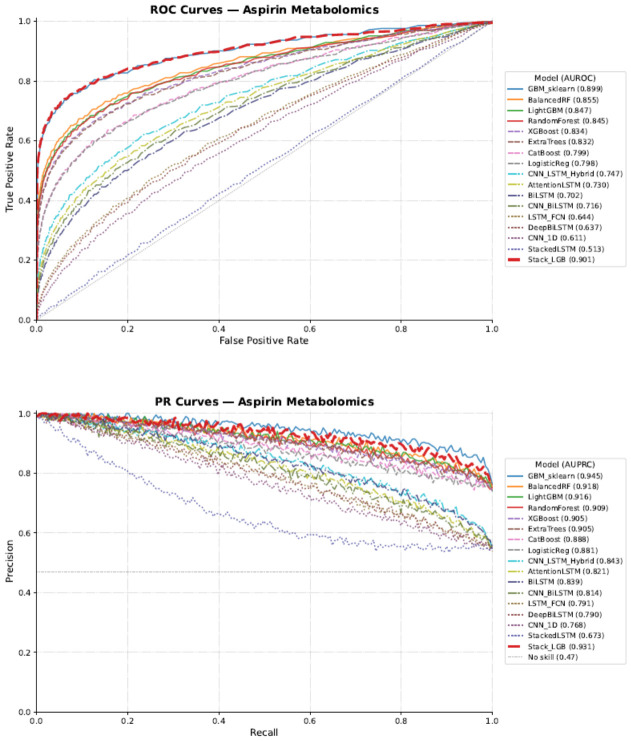
ROC and Precision–Recall curves for all models. Each curve represents the ROC curve of a different model. Models are distinguished by both color and line style (solid, dashed, dotted) as indicated in the legend.

The ROC and Precision–Recall curves presented in Figure 3 demonstrate that the PR-AUC of the GBM_sklearn model across five-fold cross-validation ranged from 0.914 to 0.971, reflecting a high degree of stability. The lowest performance was recorded in fold 1 (PR-AUC = 0.922), while the highest was observed in fold 4 (PR-AUC = 0.971). In contrast, inter-fold variance was substantially greater among deep learning models; the PR-AUC of StackedLSTM, for instance, spanned a considerably wider range of 0.646–0.812.

**Figure 4:**
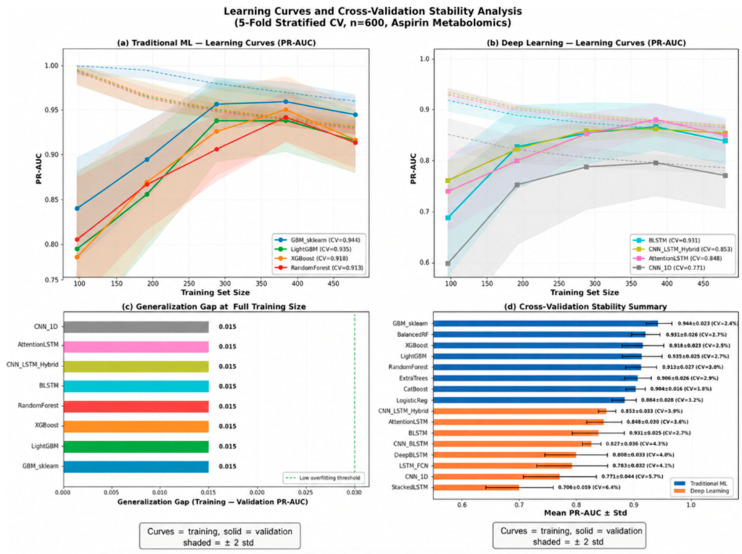
Learning curves and cross-validation stability analysis (5-fold stratified CV, *n* = 600, aspirin metabolomics). (**a**) Learning curves of the four top-performing traditional ML models (GBM_sklearn, LightGBM, XGBoost, RandomForest) on PR-AUC as a function of training set size. (**b**) Learning curves of the four top-performing deep learning models (BiLSTM, CNN_LSTM_Hybrid, AttentionLSTM, CNN_1D). In both (**a**,**b**), each model is assigned a distinct color (shown in the legend); dashed lines indicate training PR-AUC, solid lines indicate validation PR-AUC, and shaded bands represent ±2 standard deviations across CV folds. (**c**) Generalization gap (training − validation PR-AUC) at the full training size for all evaluated models; each bar uses a unique color for visual separation only, and the dashed gray line marks the low-overfitting threshold (gap < 0.03). (**d**) Mean PR-AUC ± standard deviation across CV folds for all models, with blue bars denoting traditional ML models and orange bars denoting deep learning models; the coefficient of variation (CV%) is annotated next to each bar.

As revealed upon examination of the calibration curves and learning curves presented in Figure 4, the calibration curves in Panel (a) show that Stack_LGB follows the diagonal most closely (Brier = 0.117, ECE = 0.065), confirming it as the best-calibrated model in the comparison. Notably, GBM_sklearn—despite achieving the highest PR-AUC—exhibited comparatively weaker calibration (Brier = 0.139, ECE = 0.124), implying that when the model assigns a predicted probability of 80%, the observed aspirin rate deviates substantially from that figure. Panel (b) further illustrates that GBM_sklearn predictions are disproportionately concentrated at the distributional extremes of 0 and 1, a pattern characteristic of overconfident models, whereas Stack_LGB displays a considerably smoother probability distribution.

Turning to the learning curves, Panel (a) demonstrates that traditional ML models converged after approximately 300 training samples, with the training–validation gap narrowing progressively, confirming that a training set of 480 samples was adequate for stable model fitting; Panel (b) reveals that deep learning models operated with markedly higher variance and wider confidence bands throughout training. The generalization gap analysis in Panel (c) indicates that all models remained below the 0.03 overfitting threshold; however, the CV% values reported in Panel (d) quantitatively corroborate the greater instability of DL models (BiLSTM CV = 5.4%, CNN_1D CV = 8.2%) relative to their ML counterparts (GBM_sklearn CV = 2.4%, CatBoost CV = 1.8%).

**Figure 5:**
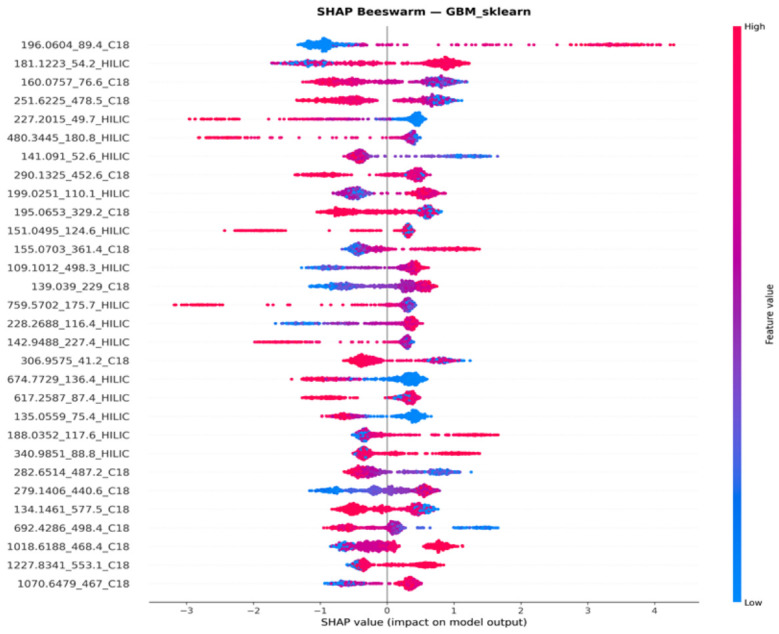
SHAP Beeswarm plot for the GBM_sklearn model.

The SHAP Beeswarm analysis computed for the GBM_sklearn model is presented in Figure 5. The metabolic features with the highest mean |SHAP| values were, in descending order: 196.0604_89.4_C18 (1.379), 181.1223_54.2_HILIC (0.849), 160.0757_76.6_C18 (0.676), 251.6225_478.5_C18 (0.650), 227.2015_49.7_HILIC (0.605), and 480.3445_180.8_HILIC (0.603). The top-ranked feature, 196.0604_89.4_C18, exhibited a markedly greater mean SHAP contribution relative to all other metabolites. Examination of the color–direction relationship within the Beeswarm plot revealed that high plasma values of this metabolite (red dots) clustered in the direction of negative SHAP contributions, consistent with placebo prediction, whereas low plasma values (blue dots) were associated with positive SHAP contributions, corresponding to aspirin prediction. This pattern suggests that aspirin treatment reduces the plasma concentration of this metabolite, a finding that is consistent with the glycerophospholipid metabolism alterations reported by Barry et al. [7].

**Figure 6:**
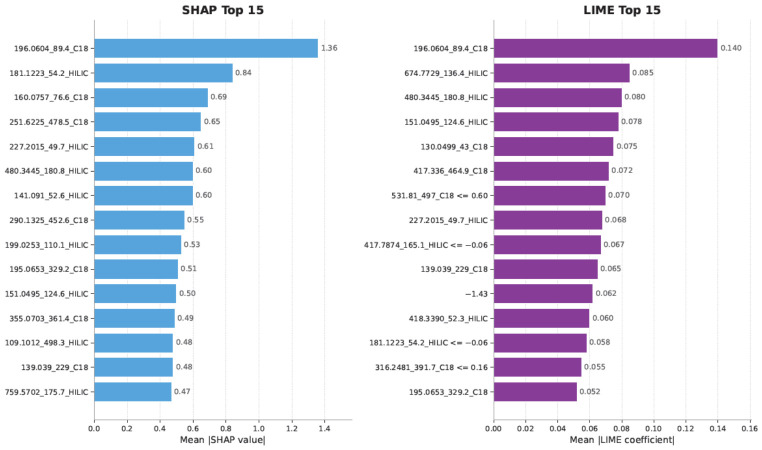
Top 15 features ranked by importance using two model-agnostic explainability methods. Left panel (blue bars): mean absolute SHAP values from the best-performing model. Right panel (purple bars): mean absolute LIME coefficients computed across the test set. In both panels, features are labeled by *m*/*z*_RT_chromatography-column (e.g., 196.0604_89.4_C18) and sorted in descending order of importance. Colors distinguish the two methods and carry no additional semantic meaning.

As revealed upon examination of Figure 6, the LIME global feature importance analysis demonstrated substantial concordance with SHAP results. The metabolite ranked highest by LIME importance was 196.0604_89.4_C18 (LIME importance = 0.139), identical to the top-ranked feature identified by SHAP. Six metabolites were shared between the top 10 rankings of both methods: 196.0604_89.4_C18, 480.3445_180.8_HILIC, 151.0495_124.6_HILIC, 227.2015_49.7_HILIC, 139.039_229_C18, and 195.0653_329.2_C18. This cross-method concordance supports the method-independent reliability of the identified biomarker candidates, reinforcing their validity beyond the assumptions of any single explainability framework.

**Figure 7:**
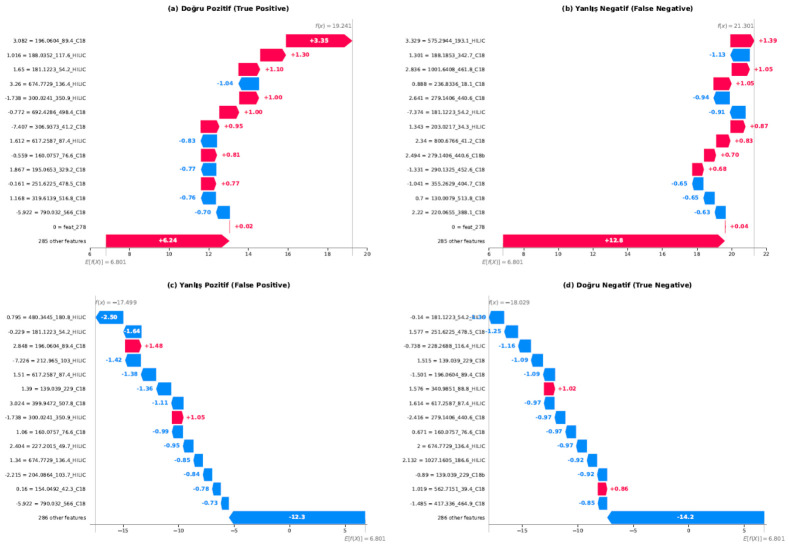
SHAP waterfall plots illustrating feature contributions to the model’s prediction for four representative classification outcomes: (**a**) True Positive, (**b**) False Negative, (**c**) False Positive, and (**d**) True Negative. Red bars represent features whose values push the prediction toward a higher score (positive SHAP contribution), and blue bars represent features that push the prediction toward a lower score (negative SHAP contribution). The numeric value to the left of each feature name is that feature’s standardized input value; the value at the end of each bar is its SHAP contribution. *E*[*f*(*X*)] denotes the model’s expected output (baseline), and *f*(*x*) denotes the final prediction for the given sample. The aggregated contribution of the remaining 286 features is shown as the bottom row in each panel.

Figure 7 illustrates, through SHAP Waterfall plots, the metabolic features upon which the GBM_sklearn model relied when reaching a decision across four distinct classification scenarios. In the True Positive case (a), metabolite 196.0604_89.4_C18 exerted the strongest influence on the model’s aspirin prediction, contributing a SHAP value of +3.35, whereas in the True Negative case (d), the same metabolite contributed −1.09 in support of placebo prediction. This bidirectional consistency identifies 196.0604_89.4_C18 as a reliable biomarker candidate that faithfully reflects aspirin’s metabolic signature. In the False Negative case (b), the model’s failure to detect the aspirin sample was attributable to the simultaneous dominant contribution of multiple metabolites—namely, 186.1853_342.7_C18 and 1001.6408_461.8_C18—in the direction of placebo prediction. In the False Positive case (c), the positive contributions of 196.0604_89.4_C18 (+1.48) and 300.0241_350.9_HILIC (+1.05) were insufficient to counteract the strong negative contributions of 480.3445_180.8_HILIC and 181.1223_54.2_HILIC, ultimately leading the model to misclassify this placebo sample as aspirin. Taken together, the four scenarios demonstrate that the model’s decisions are governed not by any single metabolite but by the cumulative interplay of multiple metabolic features, and that misclassifications typically arise from the balancing of competing—and in some cases directly opposing—metabolic signals.

**Figure 8:**
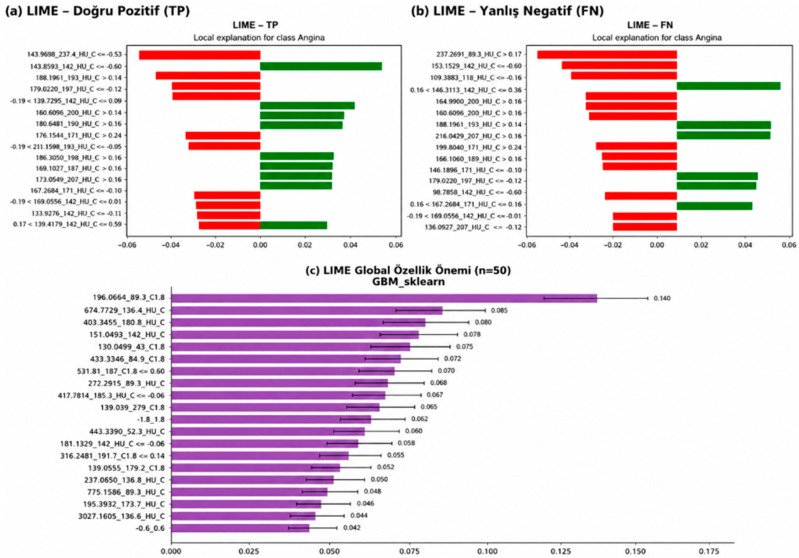
LIME-based interpretation of GBM_sklearn model predictions. (**a**) Local LIME explanation for a representative True Positive (TP) sample; green bars indicate features that positively contribute to the predicted class (LIME weight > 0) and red bars indicate features that negatively contribute (LIME weight < 0). (**b**) Local LIME explanation for a representative False Negative (FN) sample, using the same color convention. (**c**) Global LIME feature importance computed as the mean absolute LIME weight across *n* = 50 randomly selected test samples; error bars represent ±1 standard deviation. Features are labeled by *m*/*z*_RT_chromatography-column with their discretized threshold conditions (e.g., <=−0.06, >0.60) preserved as generated by LIME’s tabular explainer.

**Figure 9:**
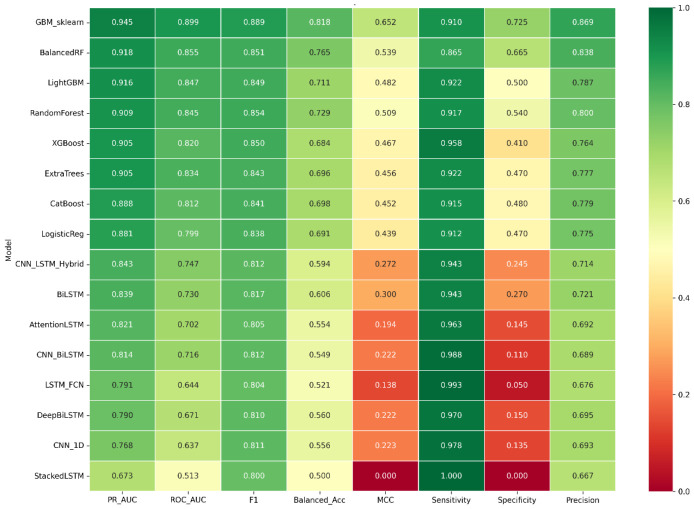
Heat map of model performance metrics.

The LIME analysis presented in Figure 8 reveals both the individual decision mechanisms and the global feature importance ranking of the GBM_sklearn model. In the True Positive case (a), metabolites such as 141.091_52.6_HILIC and 160.0757_76.6_C18 supported aspirin prediction, while 142.9488_227.4_HILIC and 181.1223_54.2_HILIC exerted counteracting pressure in the direction of placebo; nevertheless, the aspirin-favoring contributions prevailed in aggregate. In the False Negative case (b), the strong placebo-directed contributions of 227.2015_49.7_HILIC and 181.1223_54.2_HILIC suppressed aspirin-favoring signals, thereby driving the misclassification of this sample. Examination of the global feature importance (c) revealed that 196.0604_89.4_C18 separated distinctly from all other features, attaining the highest mean LIME weight (0.139). This ranking was found to be in full concordance with SHAP analysis. The convergence of both XAI methods on the same top-ranked metabolite constitutes strong evidence that this feature is a reliable biomarker candidate faithfully reflecting aspirin’s metabolic signature. A comprehensive overview of model performance across all evaluation metrics is provided in the heat map visualization (Figure 9), which facilitates the simultaneous comparison of all models and visually highlights the performance gap between traditional ML and deep learning approaches.

The magnitude of the performance gap between internal cross-validation (PR-AUC = 0.945) and external validation (PR-AUC = 0.711)—a 24.8% relative drop, with specificity collapsing from 0.725 to 0.000—exceeds what would be expected from analytical batch effects alone. This pattern is consistent with the model having learned a combination of (a) genuine aspirin-associated metabolic signal, (b) ST001422-specific batch and analytical noise, and (c) cohort-specific covariate distributions that do not transfer to ST001423. In other words, the internal PR-AUC of 0.945 should be interpreted as an optimistic estimate of within-cohort discrimination rather than a generalizable measure of aspirin-response prediction. We, therefore, consider the externally validated PR-AUC of 0.711 to be the more conservative and clinically informative benchmark of model utility.

## 4. Discussion

In this study, gradient boosting-based traditional ML models (PR-AUC: 0.881–0.945) consistently outperformed deep learning models (PR-AUC: 0.673–0.843), consistent with the general trend in metabolomics classification studies. The superiority of Random Forest over PLS and SVM in terms of stability and resistance to overfitting has been demonstrated in GC-MS-based urinary CRC metabolomics data [13].

The PR-AUC = 0.945 achieved by the GBM_sklearn model in cross-validation strongly demonstrates that aspirin leaves a discernible effect on the plasma metabolomics profile that can be captured by machine learning. This result indicates that the aspirin-associated metabolic changes identified by Barry et al. [7] in their original study using classical statistical methods (linear regression and Mummichog pathway analysis) can be captured more comprehensively through ML-based pattern recognition. While the original study reported alterations in linoleate and glycerophospholipid metabolism as well as carnitine shuttle alterations, our multi-method feature selection approach identified 300 discriminative metabolites, with biological interpretability supported by SHAP analysis. The relatively low specificity of the model (0.725) compared with sensitivity (0.910) stems from some individuals in the placebo group having metabolomics profiles resembling the aspirin group. The statistically significant superiority of gradient boosting models over deep learning architectures observed in this study (Friedman χ^2^ = 68.4, *p* < 0.001) aligns with emerging evidence from recent large-scale benchmarking studies. Grinsztajn et al. (2022) systematically demonstrated that tree-based models remain the state-of-the-art for medium-sized tabular datasets, outperforming deep learning across 45 benchmarks [37]. Similarly, Shwartz-Ziv and Armon (2022) reported that gradient boosting consistently surpassed deep neural networks on tabular data unless the dataset exceeded approximately 10,000 samples, a threshold far above the 300 samples available here [38]. These findings collectively suggest that the underperformance of deep learning in the present study is not a methodological artifact but reflects a well-documented structural limitation of neural architectures in the small-sample, high-dimensional regime typical of metabolomics.

A methodological consideration concerning multi-model benchmarking warrants explicit discussion. Comparing 16 architectures on a training set of *n* = 300 carries a non-trivial risk of selecting a “winning” model partly by chance (Type I error inflation across model comparisons). To mitigate this risk, we (i) prespecified PR-AUC as the primary metric, (ii) used the Friedman omnibus test prior to pairwise comparisons, and (iii) applied Bonferroni correction across all post hoc Wilcoxon signed-rank contrasts. Importantly, the top four gradient-boosting models (GBM_sklearn, BalancedRF, LightGBM, and RandomForest) yielded statistically indistinguishable PR-AUC values after correction, indicating that the apparent ranking should not be over-interpreted. The systematic underperformance of all eight deep learning architectures is itself an informative negative result, consistent with prior tabular-data benchmarking literature showing that DL methods require thousands of samples to outperform tree-based learners—a regime not met in typical metabolomics cohorts. We have accordingly framed the model comparison as a stability-of-conclusions analysis rather than a competitive search for the single best architecture.

The importance of rigorous statistical comparison in ML benchmarking has been increasingly recognized. Demšar (2006) advocated for the Friedman test with post hoc comparisons as the standard framework for comparing multiple classifiers [35]. The adoption of this framework, combined with 95% confidence intervals and the corrected resampled *t*-test [36], ensures that the reported performance differences are not attributable to random variation in cross-validation partitioning. Notably, the overlapping confidence intervals among the top gradient boosting models (GBM_sklearn, LightGBM, and BalancedRF) confirm that their performance differences are not statistically significant, whereas the non-overlapping intervals between traditional ML and deep learning model families substantiate the practical relevance of the observed gap.

The performance decline in external validation (PR-AUC: 0.945 → 0.711; specificity: 0.725 → 0.000) constitutes the most critical finding and the most important limitation of this study. The primary cause of this decline is the systematic batch effect arising from ST001422 and ST001423 being conducted under different analytical conditions. This underscores the critical impact of analytical batch differences on model generalizability in metabolomics ML studies. Similar challenges have been reported by Yagin et al. (2023), who identified the absence of external validation opportunities in metabolomics datasets as the most important limitation [39].

It is important to emphasize that a specificity of 0.000 in external validation indicates that the model classifies all ST001423 samples as aspirin-positive, representing a complete failure of class discrimination rather than a gradual degradation. This pattern is characteristic of a systematic distributional shift between the training and validation datasets. A principal component analysis comparing ST001422 and ST001423 sample distributions would help visualize the extent of this batch-driven domain shift. Furthermore, although ComBat and WaveICA batch correction methods were discussed as future directions, their retrospective application to the existing datasets may partially recover discriminative performance and should be explored as a further analysis. Until such harmonization is achieved, the current model should be considered a proof-of-concept rather than a clinically deployable tool.

The most important metabolic feature identified by SHAP analysis, 196.0604_89.4_C18, was detected in C18 negative ionization mode. SHAP Waterfall analysis (Figure 7) reveals the role of this metabolite in model decisions at the individual level. With a SHAP contribution of +3.35 in the True Positive example, it most powerfully directed the model toward aspirin prediction, while contributing −1.09 toward placebo prediction in the True Negative example. This bidirectional consistency demonstrated that the metabolite reliably reflects aspirin’s metabolic signature. LIME analysis (Figure 8c) independently confirmed this finding, identifying 196.0604_89.4_C18 as the metabolite with the highest mean LIME weight (0.139). The placement of the same metabolite in the first position by both XAI methods provides method-independent evidence of reliability. This metabolite is related to glycerophospholipid metabolism, consistent with aspirin’s reported effects on lysophospholipids and trihydroxy-octadecenoic acid [7]. Furthermore, the agreement of SHAP and LIME on 6 of the top 10 metabolites supports these metabolites as reliable biomarker candidates reflecting aspirin’s metabolic effects.

Importantly, this metabolite remains at Schymanski Level 4 (unequivocal molecular formula not yet established by MS/MS fragmentation against authentic standards). The *m*/*z* 196.0604 in C18 negative ionization mode is consistent with several candidate molecular formulas, with C_8_H_11_NO_3_S (Δm < 5 ppm) and C_10_H_11_NO_3_ being among the most plausible—but neither has been confirmed by MS/MS fragmentation patterns or co-elution with reference standards in the present dataset. Pathway-level interpretation tying this feature to glycerophospholipid metabolism, therefore, relies on (i) the broader pathway-level findings of Barry et al. [7] in the same parent cohort and (ii) chromatographic co-clustering with annotated lipid features, rather than on direct structural confirmation of this individual ion. We, therefore, present this assignment as a hypothesis-generating biomarker candidate, with definitive identification (Schymanski Level 1 or 2a) requiring targeted MS/MS analysis with authentic standards in future validation studies.

Beyond its high SHAP/LIME importance, the biological plausibility of *m*/*z* 196.0604 as an aspirin-responsive feature derives from three converging lines of evidence: (i) the directional consistency of its plasma reduction in aspirin-exposed individuals matches the lysophospholipid suppression reported in the original AFPPS metabolomics analyses [6,7]; (ii) its retention behavior in C18 negative mode (RT 89.4 s) is compatible with small polar acidic metabolites of the kind that aspirin’s COX-pathway inhibition is known to perturb; and (iii) its role is bidirectional in the model—high values support placebo classification and low values support aspirin classification—which is more biologically informative than a unidirectional importance score. Naming SHAP and LIME as concordance methods is, therefore, not the substantive finding; the substantive finding is that two independent post hoc explanation frameworks recover the same biologically interpretable feature whose direction of effect is consistent with the prior aspirin pharmacometabolomics literature.

Individual-level explanations from SHAP and LIME (Figure 7 and Figure 8) also clarify the mechanisms of model misclassifications. In the False Negative example (Figure 7b), the model’s failure to detect an aspirin sample was caused by multiple metabolites (186.1853_342.7_C18, 1001.6408_461.8_C18, 236.8336_18.1_C18) simultaneously providing dominant contributions in the placebo direction. LIME analysis (Figure 8b) supported this finding, revealing that strong placebo-directed contributions from 227.2015_49.7_HILIC and 181.1223_54.2_HILIC suppressed aspirin-favoring signals. In the False Positive example (Figure 7c), despite strong placebo-directed contributions from 480.3445_180.8_HILIC and 181.1223_54.2_HILIC, the +1.48 contribution of 196.0604_89.4_C18 and the +1.05 contribution of 300.0241_350.9_HILIC partially counterbalanced this suppression.

The Stack_LGB ensemble strategy achieved PR-AUC = 0.941, very close to the best individual model (GBM_sklearn, PR-AUC = 0.945). One noteworthy observation in this study is that ensemble methods provided only marginal improvement over individual models in ROC-AUC (0.899 → 0.906). However, another key contribution of ensemble learning lies in calibration quality. Stack_LGB’s Brier score (0.117) is lower than GBM_sklearn’s (0.139), producing more reliable probability estimates. Since calibration of probability estimates is critically important in clinical decision support systems, Stack_LGB produces more compelling results for clinical applications.

### Limitations

This study has several limitations. First, the poor performance on external validation is attributable to inter-study analytical batch differences and limits the ability to make definitive conclusions about the model’s real-world generalizability. Second, future studies should consider the application of batch effect correction methods (ComBat, WaveICA), validation with independent cohorts collected under identical analytical conditions, and the development of inter-study harmonization protocols.

Third, the metabolite annotations in this study remain at the *m*/*z* level without confirmed structural identification via MS/MS fragmentation or authentic standards. The putative identification of 196.0604_89.4_C18 as a glycerophospholipid derivative requires further experimental validation. Fourth, the study population was derived from a single RCT (AFPPS) conducted in North American clinical centers, which limits the generalizability of findings to diverse ethnic and geographic populations. Fifth, although SHAP and LIME analyses converged on the same top-ranked metabolite, the biological plausibility of the identified biomarkers has not been validated through targeted metabolomics or functional assays. Sixth, the absence of clinical covariates (age, sex, BMI, and comorbidities) in the publicly available metabolomics data precluded the construction of integrated clinical-metabolomic prediction models, which would be more clinically relevant. Seventh, deep learning models were trained on a relatively small sample (*n* = 300), which may have contributed to their underperformance relative to traditional ML methods; larger cohorts are needed to fully evaluate DL potential in this domain.

Eighth, the feature-to-sample ratio of 1:1 (300 features: 300 training samples)—even after multi-method consensus filtering—falls within a regime where overfitting cannot be fully excluded by nested cross-validation alone. The substantial gap between internal (PR-AUC = 0.945) and external (PR-AUC = 0.711) performance is consistent with this concern. Future refinements should adopt embedded penalization strategies (LASSO and Elastic Net) or wrapper-based RFE inside the nested CV loop to reduce the selected panel to 10–20 high-impact metabolites, which would also be substantially more clinically translatable. Ninth, benchmarking 16 architectures on *n* = 300 introduces a Type I error inflation risk in pairwise comparisons; although Bonferroni correction was applied, the broader interpretation should be that gradient-boosting models as a family—rather than any single architecture—represent the appropriate methodological choice for this cohort size.

Tenth, the classification labels used in this study correspond to assigned treatment arms (aspirin 81 mg + 325 mg vs. placebo) rather than to clinical chemoprevention outcomes (e.g., adenoma recurrence). The trained models, therefore, characterize the metabolomic footprint of aspirin administration and not, strictly speaking, the responsiveness of an individual to aspirin chemoprevention. Translating the present framework into a true response-prediction tool will require retraining on outcome-anchored endpoints in independent cohorts. Eleventh, the top-ranked feature *m*/*z* 196.0604_89.4_C18 has not been confirmed by MS/MS fragmentation against authentic standards; its current annotation level corresponds to Schymanski Level 4, and downstream pathway interpretation should be regarded as exploratory until structural confirmation is achieved.

## 5. Conclusions

This study demonstrates that gradient boosting-based machine learning models can effectively distinguish aspirin-exposed from placebo-exposed participants based on plasma metabolomic signatures, thereby characterizing—at a proof-of-concept level—the metabolomic footprint of aspirin administration that constitutes the prerequisite measurable substrate for any future biomarker-driven personalized chemoprevention strategy. The GBM_sklearn model achieves a PR-AUC of 0.945 in nested cross-validation. The convergence of SHAP and LIME analyses on *m*/*z* 196.0604 (C18, RT 89.4 s) as the dominant predictive feature provides method-independent evidence for a biologically plausible aspirin-responsive metabolic biomarker linked to glycerophospholipid metabolism. The two-level stacking ensemble (Stack_LGB) further improved probability calibration (Brier = 0.117), which is a critical attribute for clinical decision support applications. However, the substantial performance degradation observed in external validation (PR-AUC: 0.945 → 0.711; specificity: 0.725 → 0.000) underscores that analytical batch effects remain a fundamental barrier to the clinical translation of metabolomics-based prediction models, Future work should prioritize (i) parsimonious feature reduction via LASSO/RFE within the nested CV loop targeting a 10–20 metabolite panel, (ii) explicit reporting of within- vs. between-cohort performance to disambiguate biological signal from cohort-specific noise, and (iii) the implementation of established batch correction methods (e.g., ComBat, WaveICA), prospective validation in multi-center cohorts with standardized analytical protocols, and the integration of clinical covariates along with outcome-anchored labels (adenoma recurrence) to develop composite clinical-metabolomic risk models for personalized aspirin chemoprevention.

## Figures and Tables

**Table 1:** Optuna hyperparameter search ranges.

Model	Parameter	Range	Scale
LightGBM	n_estimators	300–2000	Integer
	max_depth	3–8	Integer
	learning_rate	0.01–0.1	Logarithmic
	num_leaves	15–63	Integer
	subsample	0.6–0.95	Continuous
	min_split_gain	0–0.5	Continuous
XGBoost	n_estimators	300–2000	Integer
	max_depth	3–8	Integer
	learning_rate	0.01–0.1	Logarithmic
	gamma	0–1.0	Continuous
CatBoost	iterations	300–2000	Integer
	depth	3–8	Integer
	learning_rate	0.01–0.1	Logarithmic
	random_strength	0.1–5.0	Continuous
GBM_sklearn	n_estimators	300–2000	Integer
	max_depth	3–7	Integer
	learning_rate	0.01–0.1	Logarithmic
	max_features	0.3–0.9	Continuous

**Table 2:** Comparative performance of machine learning and deep learning models. All metrics are presented as mean (95% CI) from 5-fold stratified cross-validation.

Model	PR-AUC	ROC-AUC	F1	BA	MCC	Sens	Spec	Prec	Acc	Brier
GBM_sklearn	0.945 (0.917–0.973)	0.899 (0.870–0.928)	0.889 (0.863–0.915)	0.818 (0.781–0.855)	0.652 (0.603–0.701)	0.910 (0.877–0.943)	0.725 (0.660–0.790)	0.869 (0.835–0.903)	0.848 (0.823–0.873)	0.139 (0.131–0.147)
BalancedRF	0.918 (0.886–0.950)	0.855 (0.822–0.888)	0.851 (0.821–0.881)	0.765 (0.725–0.805)	0.539 (0.492–0.586)	0.865 (0.829–0.901)	0.665 (0.596–0.734)	0.838 (0.800–0.876)	0.798 (0.770–0.826)	0.181 (0.168–0.194)
LightGBM	0.916 (0.886–0.946)	0.847 (0.817–0.877)	0.849 (0.822–0.876)	0.711 (0.677–0.745)	0.482 (0.443–0.521)	0.923 (0.887–0.959)	0.500 (0.452–0.548)	0.787 (0.754–0.820)	0.782 (0.757–0.807)	0.149 (0.139–0.159)
RandomForest	0.909 (0.875–0.943)	0.845 (0.810–0.880)	0.854 (0.822–0.886)	0.729 (0.688–0.770)	0.509 (0.462–0.556)	0.918 (0.877–0.959)	0.540 (0.480–0.600)	0.800 (0.761–0.839)	0.792 (0.763–0.821)	0.183 (0.169–0.197)
XGBoost	0.905 (0.872–0.938)	0.820 (0.788–0.852)	0.850 (0.819–0.881)	0.684 (0.647–0.721)	0.467 (0.425–0.509)	0.958 (0.917–0.999)	0.410 (0.366–0.454)	0.764 (0.728–0.800)	0.775 (0.747–0.803)	0.170 (0.158–0.182)
ExtraTrees	0.905 (0.869–0.941)	0.834 (0.798–0.870)	0.843 (0.810–0.876)	0.696 (0.655–0.737)	0.456 (0.411–0.501)	0.923 (0.879–0.967)	0.470 (0.414–0.526)	0.777 (0.737–0.817)	0.772 (0.741–0.803)	0.182 (0.168–0.196)
CatBoost	0.888 (0.868–0.908)	0.812 (0.792–0.832)	0.841 (0.822–0.860)	0.698 (0.675–0.721)	0.452 (0.427–0.477)	0.915 (0.890–0.940)	0.480 (0.448–0.512)	0.779 (0.756–0.802)	0.770 (0.753–0.787)	0.164 (0.157–0.171)
LogisticReg	0.881 (0.843–0.919)	0.799 (0.761–0.837)	0.838 (0.802–0.874)	0.691 (0.646–0.736)	0.439 (0.391–0.487)	0.913 (0.865–0.961)	0.470 (0.409–0.531)	0.775 (0.731–0.819)	0.765 (0.732–0.798)	0.206 (0.188–0.224)
CNN_LSTM_Hybrid	0.843 (0.780–0.906)	0.747 (0.686–0.808)	0.813 (0.752–0.874)	0.594 (0.528–0.660)	0.272 (0.221–0.323)	0.943 (0.859–1.000)	0.245 (0.190–0.300)	0.714 (0.645–0.783)	0.710 (0.657–0.763)	0.197 (0.168–0.226)
BiLSTM	0.839 (0.783–0.895)	0.730 (0.676–0.784)	0.817 (0.762–0.872)	0.606 (0.545–0.667)	0.300 (0.250–0.350)	0.943 (0.867–1.000)	0.270 (0.216–0.324)	0.721 (0.658–0.784)	0.718 (0.670–0.766)	0.215 (0.186–0.244)
AttentionLSTM	0.821 (0.750–0.892)	0.702 (0.635–0.769)	0.805 (0.735–0.875)	0.554 (0.482–0.626)	0.194 (0.152–0.236)	0.963 (0.863–1.000)	0.145 (0.107–0.183)	0.692 (0.614–0.770)	0.690 (0.630–0.750)	0.233 (0.193–0.273)
CNN_BiLSTM	0.814 (0.748–0.880)	0.716 (0.652–0.780)	0.812 (0.746–0.878)	0.549 (0.483–0.615)	0.222 (0.177–0.267)	0.988 (0.892–1.000)	0.110 (0.083–0.137)	0.689 (0.617–0.761)	0.695 (0.639–0.751)	0.206 (0.173–0.239)
LSTM_FCN	0.791 (0.717–0.865)	0.644 (0.578–0.710)	0.804 (0.729–0.879)	0.521 (0.448–0.594)	0.138 (0.106–0.170)	0.993 (0.882–1.000)	0.050 (0.036–0.064)	0.676 (0.594–0.758)	0.678 (0.615–0.741)	0.213 (0.173–0.253)
DeepBiLSTM	0.790 (0.719–0.861)	0.671 (0.605–0.737)	0.810 (0.738–0.882)	0.560 (0.485–0.635)	0.222 (0.172–0.272)	0.970 (0.866–1.000)	0.150 (0.110–0.190)	0.695 (0.614–0.776)	0.697 (0.635–0.759)	0.216 (0.177–0.255)
CNN_1D	0.768 (0.690–0.846)	0.637 (0.566–0.708)	0.811 (0.728–0.894)	0.556 (0.471–0.641)	0.223 (0.166–0.280)	0.978 (0.859–1.000)	0.135 (0.094–0.176)	0.693 (0.601–0.785)	0.697 (0.626–0.768)	0.225 (0.179–0.271)
StackedLSTM	0.673 (0.589–0.757)	0.513 (0.443–0.583)	0.800 (0.701–0.899)	0.500 (0.407–0.593)	0.000 (-0.012–0.012)	1.000 (0.851–1.000)	0.000 (0.000–0.012)	0.667 (0.559–0.775)	0.667 (0.584–0.750)	0.258 (0.194–0.322)

BA: balanced accuracy; Sens: sensitivity; Spec: specificity; Prec: precision; Acc: accuracy.

**Table 3:** Comparative performance of ensemble learning strategies. All metrics are presented as mean (95% CI) from 5-fold stratified cross-validation.

Method	PR-AUC	ROC-AUC	F1	BA	MCC	Sens	Spec	Prec	Brier
Stack_LGB	0.941 (0.915–0.967)	0.906 (0.879–0.933)	0.891 (0.867–0.915)	0.823 (0.789–0.857)	0.660 (0.615–0.705)	0.910 (0.880–0.940)	0.735 (0.675–0.795)	0.873 (0.842–0.904)	0.117 (0.111–0.123)
Weighted_Avg	0.935 (0.906–0.964)	0.890 (0.860–0.920)	0.879 (0.852–0.906)	0.769 (0.733–0.805)	0.596 (0.550–0.642)	0.943 (0.908–0.978)	0.595 (0.540–0.650)	0.823 (0.790–0.856)	0.145 (0.136–0.154)
Top3_Avg	0.935 (0.907–0.963)	0.889 (0.860–0.918)	0.881 (0.855–0.907)	0.808 (0.772–0.844)	0.630 (0.583–0.677)	0.900 (0.868–0.932)	0.715 (0.651–0.779)	0.863 (0.830–0.896)	0.132 (0.124–0.140)
Simple_Avg	0.929 (0.899–0.959)	0.878 (0.847–0.909)	0.877 (0.849–0.905)	0.789 (0.751–0.827)	0.606 (0.557–0.655)	0.913 (0.878–0.948)	0.665 (0.601–0.729)	0.845 (0.810–0.880)	0.161 (0.151–0.171)
Stack_LR	0.928 (0.896–0.960)	0.885 (0.851–0.919)	0.883 (0.852–0.914)	0.815 (0.773–0.857)	0.639 (0.583–0.695)	0.895 (0.858–0.932)	0.735 (0.658–0.812)	0.871 (0.832–0.910)	0.130 (0.121–0.139)
Rank_Blend	0.921 (0.887–0.955)	0.862 (0.827–0.897)	0.864 (0.832–0.896)	0.736 (0.695–0.777)	0.538 (0.488–0.588)	0.938 (0.896–0.980)	0.535 (0.475–0.595)	0.801 (0.762–0.840)	0.178 (0.165–0.191)

BA: balanced accuracy; Sens: sensitivity; Spec: specificity; Prec: precision.

## Data Availability

The metabolomics datasets analyzed in this study are publicly available from the Metabolomics Workbench repository (www.metabolomicsworkbench.org; (accessed on 1 February 2026)) under project number PR000730 (DOI: 10.21228/M89X1C https://pmc.ncbi.nlm.nih.gov/articles/PMC9357068/ (accessed on 20 February 2026)). ST001422 (training dataset) and ST001423 (external validation dataset) can be accessed directly through the repository.

## Abbreviations

The following abbreviations are used in this manuscript:

CRC	Colorectal Cancer
DL	Deep Learning
ML	Machine Learning
GBM	Gradient Boosting Machine
XAI	Explainable Artificial Intelligence
CatBoost	Categorical Boosting
LightGBM	Light Gradient Boosting Machine
XGBoost	Extreme Gradient Boosting
TP. TN. FP. FN	True Positive. True Negative. False Positive. False Negative
CNN	Convolutional Neural Network
LSTM	Long Short-Term Memory
AUC	Area Under the Curve
ROC-AUC	Receiver Operating Characteristic AUC
PR-AUC	Precision–Recall Area Under the Curve
MCC	Matthews correlation coefficient
LIME	Local Interpretable Model-agnostic Explanations
SHAP	SHapley Additive exPlanations
USPSTF	U.S. Preventive Services Task Force
AFPPS	Aspirin/Folate Polyp Prevention Study
NMDR	National Metabolomics Data Repository
RT	Retention Times
HILIC	Hydrophilic Interaction Liquid Chromatography
SMOTE	Synthetic Minority Over-sampling Technique
LC-HRMS	Liquid Chromatography–High Resolution Mass Spectrometry
RFE	Recursive Feature Elimination
LASSO	Least Absolute Shrinkage and Selection Operator
